# COVID-19 and social distancing: pandemic has altered social relationships and contacts in older adults over 4 years

**DOI:** 10.3389/fpubh.2024.1456829

**Published:** 2024-12-16

**Authors:** Lydia Kastner, Ulrike Suenkel, Anna-Katharina von Thaler, Gerhard W. Eschweiler, Theresa Dankowski, Christian Mychajliw, Kathrin Brockmann, Sebastian Heinzel, Ansgar Thiel

**Affiliations:** ^1^Institute for Sport Science, Eberhard Karls University of Tübingen, Tübingen, Germany; ^2^Tübingen Center for Mental Health (TüCMH), Department of Psychiatry and Psychotherapy, Tübingen University Hospital, Tübingen, Germany; ^3^German Center for Mental Health (DZPG), Partner Site Tübingen, Tübingen, Germany; ^4^Department of Neurology, University Medical Centre Schleswig-Holstein and Kiel University, Kiel, Germany; ^5^Department of Neurodegeneration, Hertie Institute for Clinical Brain Research, University of Tübingen, Tübingen, Germany; ^6^Geriatric Center, Tübingen University Hospital, Tübingen, Germany; ^7^Institute of Medical Informatics and Statistics, University Medical Centre Schleswig-Holstein and Kiel University, Kiel, Germany; ^8^German Center for Neurodegenerative Diseases, University of Tübingen, Tübingen, Germany; ^9^LEAD Graduate School and Research Network, Eberhard Karls University of Tübingen, Tübingen, Germany; ^10^German Sport University Cologne (DSHS), University Cologne, Cologne, Germany

**Keywords:** COVID-19, social contact, social networks, loneliness, older adults

## Abstract

**Introduction:**

Social isolation is a main risk factor for loneliness, health issues and psychological diseases. With its restriction measures, the coronavirus pandemic has led to an objective reduction in meaningful interactions, communication, and social contacts in general (social isolation). However, it has been shown that older adults cope differently with social isolation. Therefore, the aim of the present study was to investigate the changes of social contacts of older adults over the pandemic period of 4 years.

**Methods:**

For this purpose, *N* = 175 older adults (*M*_age_ = 72.60, *SD*_age_ = 6.12 years, *Mdn*_age_ = 72, Range: 60–87 years) were asked at 3 time points (2019, 2021, 2023) with how many people they had contact in the reference month (May, November). In addition to the number of contacts, participants were also asked about the type of the relationship (e.g., family, friends, neighbors), the type of contact (e.g., telephone, video conference and/or by written messages) and the emotional closeness (close, medium, low). We used an ego-centered “social network” circle to measure social contacts of older adults before, during and after the pandemic. The data collection was limited by the changing corona restrictions.

**Results:**

Results indicate that behavior in social contacts essentially depends on age, gender, and level of depression. We found a clear temporal drop in social contacts independently of age and gender during the pandemic. After the pandemic close contacts did not recover to prepandemic level. Especially, Young-Old (<72 years) recovered less in terms of the number of social contacts than the Old-Old (≥72 years).

**Discussion:**

Our study, thus, provides longitudinal insights into the course of social contacts and suggests that social isolation may have more negative and long-term impact on close contacts, which need further clarification and temporal extension.

## 1 Introduction

The outbreak of the COVID-19 pandemic in late 2019 heralded an era of unprecedented global disruption, impacting various aspects of daily life for people of all ages (1–3). Among the population groups particularly affected by the pandemic are older adults facing particular challenges in terms of their age-related frailty and their increased susceptibility to serious illnesses caused by the coronavirus [SARS-CoV-2; (1, 4)]. Governments, therefore, worldwide introduced strict restrictions such as physical distancing, lockdowns, and quarantine protocols to contain the spread of the virus (5, 6). Due to these restrictions (older), people experienced “side effects” of the pandemic in terms of higher levels of stress, anxiety, depression, and loneliness (so-called *psychological* consequences). In addition, they had limited access to health services; daily routines and activities were disrupted and a lot of them had a lack of exercises (*physical* consequences) and social contacts [*sociological* consequences, (7–9)].

Up to now, the world continues to struggle with the ongoing consequences of the pandemic and therefore the government is interested to investigate these long-term effects [e.g., (10)]. For instance, the German loneliness barometer (published in 05/2024) makes statements on the development of loneliness in Germany, identifying vulnerable groups, risk factors and trends in the burden of loneliness and comparing them with other countries. They are also interested in which factors might be important regarding loneliness, such as the *type of the relationship* (e.g., family, friends, professionals), *type of contact* (e.g., telephone, video conference and/or by written messages) and the *emotional closeness* (close, medium, low) to individuals.

However, the relationship between participation in social contacts/isolation from social contacts and loneliness is not new (11–13). *Loneliness* is defined as a significant risk factor for several mental illnesses [e.g., (14)], and is understood as the subjective feeling of being alone (12, 15). People who feel alone are not only aware of their distance from other people, they also long for fulfilling relationships (15). In contrast, *social isolation* is primarily defined as a state characterized by an objective lack of meaningful communication and social contacts (12, 15). However, the COVID-19 pandemic in particular has increased the experience of loneliness in society due to the objective lack of important social contacts. Many research articles, therefore, already addressed the fact that the pandemic has led to changes in social contacts for many people and in the form in which contact takes place (16, 17). In particular, there is little research addressing the long-term impact of social isolation, social contacts and the role of socioeconomic factors such as age, gender, and the form and quality of social contact on older adult's health wellbeing during a pandemic.

### 1.1 Study aims

The present study therefore attempts to fill this gap by conducting an *explorative analysis* to shed light on the impact of social contacts and socioeconomic variables on older adults' contact behavior during the COVID-19 pandemic. By examining these factors, we were interested in the long-term changes in social contacts during the COVID-19 pandemic and the association with depression in a cohort of older adults who have been participating in a longitudinal cohort study since long before the pandemic. The aim of our study was to investigate changes in older adults' social contacts and possible associations with depression during the four-year pandemic period (2019 to 2023). Our approach was exploratory, and these are the first results of a series of further planned data analyses.

## 2 Methods and materials

### 2.1 Participants

The data presented in this research article were collected from participants of a longitudinal cohort study (***T***übingen Evaluation of ***R***isk Factors for ***E***arly Detection of ***N***euro***D***egeneration, TREND, http://www.trend-studie.de), that has been running since 2009. The aim of the TREND study is to improve the early detection of Parkinson's disease and dementia. Originally, 1,201 healthy older adults (50+) from southern Germany were recruited for TREND. The cohort includes participants with an increased risk of neurodegeneration (e.g., due to hyposmia, depression, REM sleep behavior disorder or relatives with Parkinson's disease or dementia), control subjects without these risk factors or prodromal markers and participants of a previous early detection study for Parkinson's disease [PRIPS, (18, 19)]. For more information about the original sample and study see the inclusion/exclusion criteria (18). Participants undergo a comprehensive assessment (including neuropsychological testing, movement measurement, questionnaires) in on-site visits at 2-year intervals. TREND is currently in its 6th follow-up. The TREND study complies with the guidelines for good scientific practice of the Declaration of Helsinki (1964) and its later amendments and the University of Tübingen (Germany). The study received approval from the local ethics committee of the University Hospital of Tübingen (No. 90/2009BO2). All participants provided their written consent to take part in the study.

In 03/2020, the regular TREND on-site visits had to be paused due to the restrictions of the COVID-19 pandemic to reduce the risk our participants becoming infected with SARS-CoV-2 (5, 6). The pandemic has raised new research questions about our cohort, such as how this cohort of older people (average age at the start of the pandemic was around 74) is coping with the acute and long-term effects of the pandemic (known as “side effects”), particularly the impact of self-imposed or government-imposed restrictions on social contact. Since May 2020, 807 participants of the TREND study have voluntarily taken part at least once in additional surveys to answer research questions in the context of the COVID-19 pandemic.

The collection of data on social networks began in July 2021. A total of 217 TREND participants took part in this voluntary additional survey in 2021/2022; 175 of those (*M*_age_ = 72.60, *SD*_age_ = 6.12 years, *Mdn*_age_ = 72, Range: 60–87 years) also in 2023 (dropout rate: *n* = 42; 19.4%). We primarily recruited participants who had already completed the 5th follow-up of the TREND study before the pandemic. As the assessments were associated with increased effort for the participants and did not directly serve the TREND study objective, it was mainly cognitively fitter participants who were willing and able to take part in these additional surveys.

### 2.2 Method for surveying the participant's social networks

To investigate the social contacts of our participants, we used a sociological method, specifically ego-centered social networks (19–22). Participants were asked at three time points “before the pandemic” (2019, retrospective), “during the pan-demic” (2021) and “after the pandemic” (2023) about their social contacts in a specific reference month (May, November). The first two time points (before, during) were recorded at the same time.

The data was collected in face-to-face study visits (July 2021 to November 2021, *n* = 79, 6% female) or by video conference (23) (December 2021 to March 2022, *n* = 96, 35% female) for the time points “before the pandemic” and “during the pandemic”. This initially not planned switch from face-to-face to video conferencing became necessary due to the renewed tightening of restrictions and regulations by the government during the Omicron wave. The data for the third time point “after the pandemic” was collected by mail post, without face-to-face or video contact with the participants.

In order to avoid seasonal effects of social contacts, we defined two reference months (May, November). Participants who took part between July and November 2021 (*n* = 60) had May 2021 as reference month; while those who took part between December 2021 to March 2022 (*n* = 115) had November 2021 as reference. For the time point “before the pandemic” (2019) the same reference month as in 2021 was used.

**Figure 1:**
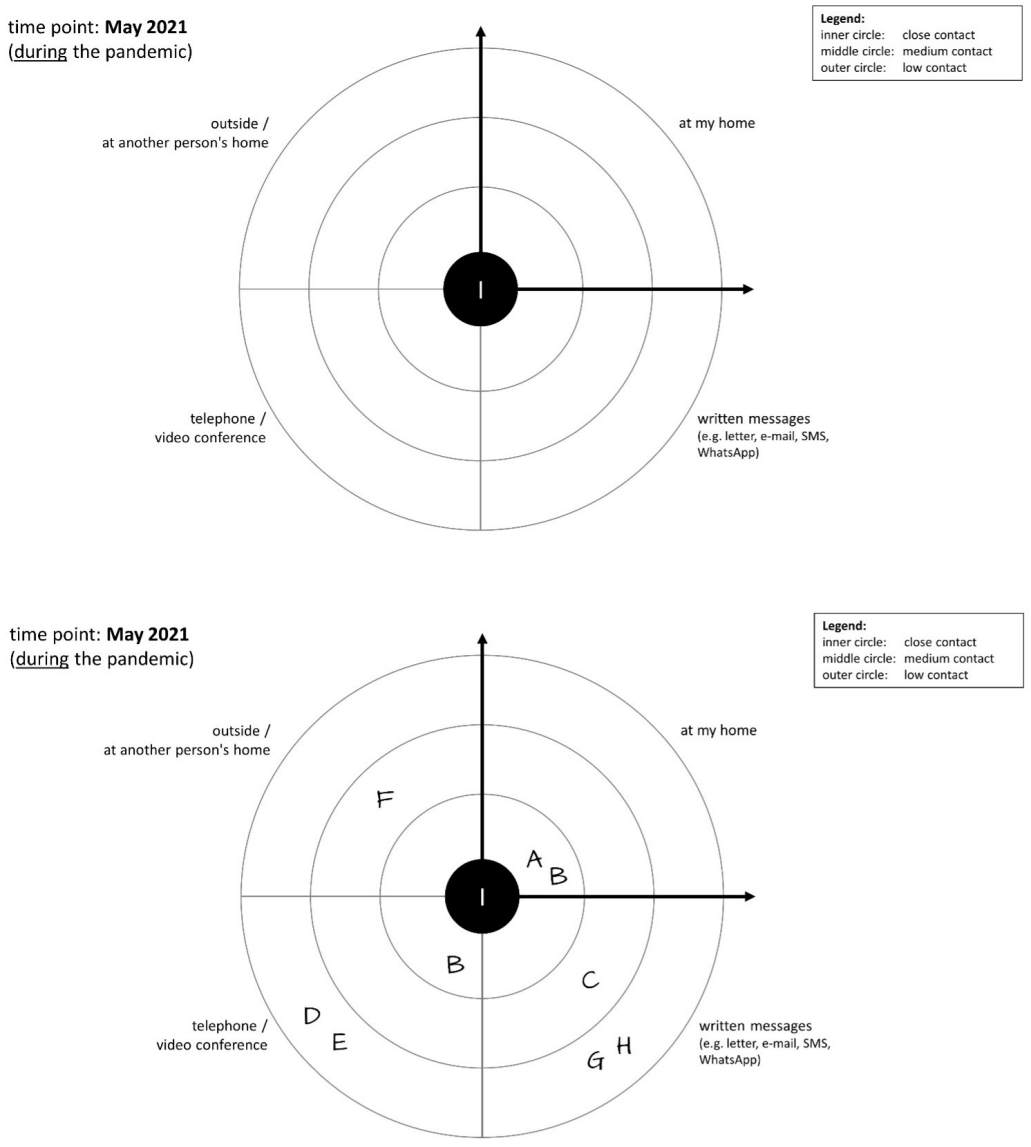
Social network circle for measuring the (1) type of relationship, (2) type of contact, and (3) emotional closeness. **(Top)** Blank scheme for the social network that is given to the participants to enter their contacts, here for the reference month of May 2021. **(Bottom)** Example of a completed scheme of a participant who indicated a total of 8 contact persons (A to H). Of these, two contacts are close contacts (A and B), two are medium contacts (C and F) and four are low contacts (D, E, G, and H). Furthermore, contact with two persons took place at the participant's own home, with three persons there was contact via written messages, there was telephone contact with three persons and one person was met outside or at a location outside the participant's own home. Using information that the investigator noted down on an additional list during the data collection (see Supplementary material), it is also possible to calculate how many of the contact persons A to H belong to the participant's family, are friends/acquaintances, neighbors, (former) work colleagues, professional helpers or social contacts in the context of voluntary work or leisure activities.

The data for the time points “before the pandemic” and “during the pandemic” was collected as follows: First, under the supervision and guidance of an investigator, the participants had to fill out a list of their contact persons who were part of their social network in the reference month 2021 (“during the pandemic”) (see Supplementary material S1). The contact persons were assigned letters (A to Z3). For the subsequent collection of information on the closeness and type of contact, we used a concentric circle diagram, similar to other egocentric network survey methods, in which the center of the circle symbolizes the participant (see Figure 1). The concentric circles form three areas of *emotional closeness* in which the participants can place their contact persons: close, medium and low contact. The closer a contact person is placed to the center of the circle, the closer the emotional contact between the participant and this person. Furthermore, the circle is divided into quarters; these areas are used to specify the *types of contact* with a person (at one's own home, via written messages, phone calls, or outside one's own home/outdoors). Participants were instructed to place each contact person in the best fitting position in the circle diagram.

After completion of the 2021 social network, the participants were asked to think back to the reference month 2 years earlier (“before the pandemic”, 2019) and fill in a second circle diagram, analogous to the 2021 diagram. During the process, additional contact persons could be added to the list, e.g., persons who died before 2021 or with whom there was contact in 2019 but no longer during the pandemic.

In addition, the investigator asked the participants about the *type of relationship* with each contact person [family, friends, neighbors, (former) colleagues, professional helpers, club/association/initiative, leisure activities (hobbies, sports) and others] and made a note of this on another form (see Supplementary material S1, p. 6 ff).

The study visits for the collection of social network data lasted approx. 1–2 h per participant.

With this method of data collection, the contact persons of a participant could be categorized in three dimensions: (1) *type of relationship* [family, friends, neighbors, (former) colleagues, professional helpers, club/volunteer work, leisure activities (hobbies, sports), and others], (2) *type of contact* (in the participant's home, through written communication, by phone/video conference, and/or outside the participant's home/outdoors), and (3) *emotional closeness* (close, medium, low).

For the third time point “after the pandemic” (2023), for economic reasons it was not possible to collect the data of the participant's social networks in the same way as in 2021 (face-to-face or by video). Therefore, the data was collected by mail using a highly individualized questionnaire (see Supplementary material) for each participant.

In early December 2023, the participants received written instructions and a personalized questionnaire in which all previously named contact persons were listed. For each of these persons, the participants were asked to indicate if the person still belonged to their social network in November 2023. If so, participants were asked how close they were to this person (single choice) and in what way they had contact with this person (multiple choice). It was possible to add new contact persons who were not part of a participant's social network in 2019 or 2021. In this way, the questionnaire covers the same three *dimensions (type of relationship, type of contact and emotional closeness)* for each contact person that were used for the previous two time points. In pilot tests (*n* = 2), it proved to be easy for our participants (even with a diagnosis of mild cognitive impairment) to complete the questionnaire without supervision. The participants were offered support by e-mail or telephone if needed, but this was rarely requested.

The results appeared plausible in comparison with the data previously collected in a different way. A total of 217 participants from the TREND cohort took part in 2021/2022 (“before the pandemic” and “during the pandemic”); in 2023, 175 participants completed the postal survey for the time point “after the pandemic”.

#### 2.2.1 Data entry and calculation of network variables

For data digitalization, we used the electronic data acquisition tool REDCap of the University of Tübingen (24). Data was entered for each contact person for each of the three points in time in the three dimensions mentioned above *(“emotional closeness”, “type of contact”, “type of relationship”)*. To calculate the social network variables for each of the three time points, the raw social network data was downloaded from RedCap and reorganized using Microsoft EXCEL (25). This made it possible to calculate not only the total number of social contacts for each participant for all three time points, but also the numbers of social contacts for all the above mentioned categories and combinations of these categories; e.g., the number of social contacts with whom there was close contact, or the number of social contacts with whom contact was maintained by telephone, or the number of social contacts who were family members. Figure 1 shows an example of a social network; the figure caption describes the calculation of the numbers in this example.

For the variables used in the data analyses for this article, the calculation was done as follows:

- **Total number of contacts:** All contact persons of a participant in the reference month were counted.
- **Number of close contacts:** All contact persons who were placed in the inner circle were counted.
- **Number of medium contacts:** All contact persons who were placed in the middle circle were counted.
- **Number of low contacts:** All contact persons who were placed in the outer circle were counted.

Sometimes groups (e.g., running group, choir) were listed as “contact persons”. In this case, the number of group members was used for the calculations.

With these numbers, it is possible to analyze whether there is an increase or decrease in social contacts in relation to the total number of contacts or a change in the number of close, medium or low contacts.

### 2.3 Psychosocial variables

Depression, loneliness, health-related quality of life, perceived social support, perceived stress, and physical (in)activity were assessed by postal or online questionnaires (05/2020 to 11/2023). We matched the questionnaire data with the periods to which the social networks refer (May and November in the respective years). For the time before the pandemic, data from the last regular TREND study visit before the start of the pandemic was used. For a more detailed description of the questionnaires used, see Table 1.

**Table 1:** Description of covariates and questionnaires.

**Questionnaire**	**Description**
Depression	To measure severity of depression, the *Becks Depression Inventory* (BDI) was used as self-report questionnaire. It was developed in the USA in 1961, revised in 1978 (40, 41); the latest German translation and validation for the BDI-I (28). Since 1996, there has been a newer version adapted to DSM-IV [BDI-II, (42)] for which the latest German translation and validation used in TREND is from 2009 (43). Participants had to choose one of four statements which they mostly described their feelings and behavior in the last 2 weeks. Thereby, 0–13 scores indicate minimal depression, 14–19 mild depression, 20–28 moderate depression and 29–63 severe depression. Scores ≥14 are referred to as clinically relevant depression.
Loneliness	We used a 6-item questionnaire (44, 45) to measure overall loneliness. Participants were asked to indicate on a 4-point Likert scale how much they agree with the statements personally (*not at all true* to *true exactly)* in the last 3 months (example-item: “*I miss people who make me feel good”*). Total scores range from 0 (*not lonely at all*) to 6 (*very lonely*).
Health-related quality of life	To measure health-related quality of life, the visual analog self-report scale from the EQ-5D-5L (46) was used with endpoints labeled “*The worst health you can imagine”* (0) and “*The best health you can imagine”* (100 scores).
Perceived stress	Stress was assessed using the 10-item *Perceived Stress Scale* (47). Participants were asked how often they felt stressed in the last month (example-item: “*In the last month, how often have you been upset because something unexpected happened?”*, answer options: *never, almost never, sometimes, quite often, very often*). The total score ranges from 0 (*no perceived stress*) to 40 points (*very strong perceived stress*).
Perceived social support	Perceived social support was measured using the F-SozU K-6 (48), which is a short form of the F-SozU [Fragebogen zur Sozialen Unterstützung; Social Support Questionnaire, (49)]. Participants were asked to indicate on a 5-point Likert scale from 1 *(strongly disagree)* to 5 *(strongly agree)* how much statements such as “*When I am sick, I can ask friends/relatives to handle important things for me without hesitation.”* currently apply to them. Total scores range from 1 *(very low perceived social support)* to 5 *(very high perceived social support)*.
Physical (in)activity	A question from the German National Health Interview and Examination Survey was used to assess physical (in)activity: “*How often do you exercise?”* meaning activities with increased heart-rate or sweating, with the answer options “*no activity”*, “* < 1 h (hrs)/week”*, “*1–2 h/week”*, “*2–4 h/week*”, and “*>4 h of physical activity per week”* [ordinal data] (50).

### 2.4 Analytical approach

We performed the data analyses using the lme4 package (26) in the free software R (27). For the social network analyses, we analyzed the number of social contacts (social contact_total_) using a generalized mixed effects model (GLMM) with Poisson distribution. Since we found that our dependent variable social contact_total_ was right skewed, with higher frequency of observations at lower values and a long tail extending toward higher values. This deviation from normality violated the assumptions of traditional linear regression models. To investigate the effects on social contact_total_, we used a GLMM with random intercepts for participants and fixed effects for time point of the pandemic (before, during, after), depression, age, and gender. For our analysis of the social contact_total_, we excluded the top 5% percentile of social contact_total_ (>120 social contacts). A total of six participants were excluded. Results were considered statistically significant when rejected alpha at *p* < 0.05.

## 3 Results

All reported data as well as the analysis script can be found in the Supplementary material. For the analyses, 175 participants with complete social networks (before, during and after the pandemic) were included in the analysis. For analyzing the changes in the social network over time, a generalized mixed effects model (GLMM) with a Poisson distribution was used (as described in the Analytical Approach).

### 3.1 Demographic and psychosocial data

To investigate whether the participants differ regarding their demographic (age, years of education) and psychosocial data [subjective reported depression level, loneliness, health-related-quality of live, perceived social support, perceived stress, and physical (in)activity] at the three time points (before/2019, during/2021, after/2023 the pandemic) and reference months, we conducted separate analyses of variance. Table 2 shows the means and standard deviations at the three time points and the two reference months. Results indicate no differences between the reference months, except age, depression, loneliness, perceived stress, and social contact_low._. Results indicate, as expected differences between the 3 time points (see Table 2).

**Table 2:** Means (M) and standard deviations (SD) for the different sociodemographic variables and time points (before, during, after) of the pandemic and reference months (May, November).

	**Time point**						
	**Before the pandemic (“2019”)**		**During the pandemic (“2021”)**		**After the pandemic (“2023”)**		
	***M***	***SD***	***M***	***SD***	***M***	***SD***	***F-statistic, p-value***
Age	71.87	5.99	73.80	5.92	76.42	5.92	*F*_Month_**(1, 519)** **=** **25.40**, ***p*** **<** **0.001**
	68.54	6.07	71.54	6.09	73.60	6.10	*F*_Time_**(2, 519)** **=** **28.69**, ***p*** **<** **0.001**
							*F*_Month × *Time*_(2, 519) = 0.31, *p* = 0.732
Depression (BDI-II)	7.25	7.87	10.38	8.98	9.88	9.02	*F*_Month_**(1, 516)** **=** **20.17**, ***p*** **<** **0.001**
	4.83	5.63	6.82	6.79	6.96	6.32	*F*_Time_**(2, 516)** **=** **6.21**, ***p*** **=** **0.002**
							*F*_Month × *Time*_(2, 516) = 0.25, *p* = 0.778
Loneliness	1.08	1.69	1.64	1.59	0.98	1.64	*F*_Month_**(1, 513)** **=** **8.59**, ***p*** **=** **0.004**
	0.69	1.33	1.10	1.49	0.71	1.39	*F*_Time_**(2, 513)** **=** **5.74**, ***p*** **=** **0.003**
							*F*_Month × *Time*_(2, 513) = 0.33, *p* = 0.719
Health-related quality of life	75.63	14.26	72.55	18.67	73.32	16.17	*F*_Month_(1, 519) = 2.31, *p* = 0.130
	78.30	12.64	73.98	17.97	76.02	17.30	*F*_Time_(2, 519) = 2.52, *p* = 0.082
							*F*_Month × *Time*_(2, 519) = 0.08, *p* = 0.926
Perceived stress	–	–	13.25	7.65	13.20	10.82	*F*_Month_**(1, 345)** **=** **5.77**, ***p*** **=** **0.017**
	–	–	11.88	6.18	10.82	6.80	*F*_Time_(1, 345) = 0.93, *p* = 0.336
							*F*_Month × *Time*_(1, 345) = 0.42, *p* = 0.517
Perceived social support	–	–	3.98	0.88	4.34	0.95	*F*_Month_(1, 345) = 2.81, *p* = 0.094
	–	–	4.18	0.78	4.30	0.72	*F*_Time_(1, 345) = 2.74, *p* = 0.099
							*F*_Month × *Time*_(1, 345) = 0.27, *p* = 0.606
Physical (in)activity	2.49	1.24	2.73	1.29	2.53	1.23	*F*_Month_(1, 516) = 3.57, *p* = 0.059
	2.36	1.29	2.27	1.20	2.48	1.21	*F*_Time_(2, 516) = 0.28, *p* = 0.757
							*F*_Month × *Time*_(2, 516) = 1.20, *p* = 0.302
Contact_total_	49.93	39.08	30.92	32.01	48.05	36.01	*F*_Month_**(1, 519)** **=** **9.60**, ***p*** **=** **0.002**
	56.82	41.65	50.04	33.12	53.77	38.17	*F*_Time_**(2, 519)** **=** **4.16**, ***p*** **=** **0.016**
							*F*_Month × *Time*_(2, 519) = 1.56, *p* = 0.208
Contact_close_	14.52	30.54	7.85	8.49	7.13	8.47	*F*_Month_(1, 519) = 0.09, *p* = 0.767
	10.36	10.65	9.70	7.83	8.36	9.02	*F*_Time_**(2, 519)** **=** **3.83**, ***p*** **=** **0.022**
							*F*_Month × *Time*_(2, 519) = 2.41, *p* = 0.091
Contact_medium_	17.28	21.30	11.50	29.03	19.62	31.64	*F*_Month_(1, 519) = 1.47, *p* = 0.226
	18.28	16.29	17.77	16.75	19.66	20.96	*F*_Time_(2, 519) = 1.49, *p* = 0.225
							*F*_Month × *Time*_(2, 519) = 0.93, *p* = 0.395
Contacts_low_	18.13	23.18	11.57	14.55	21.30	25.23	*F*_Month_**(1, 519)** **=** **11.98**, ***p*** **=** **0.001**
	28.19	33.38	22.57	23.29	25.76	29.66	*F*_Time_(2, 519) = 2.66, *p* = 0.071
							*F*_Month × *Time*_(2, 519) = 0.69, *p* = 0.501

Physical (In)activity: participants were asked how often they do sports? [1] >4 h/week, [2] 2–4 h/week, [3] 1–2 h/week, [4] < 1 hs/week, [5] no sports. Upper row shows reference month May, lower row reference month November. The bold values describe the statistical significance, *p* < 0.05.

### 3.2 Results of the social network analysis

**Figure 2:**
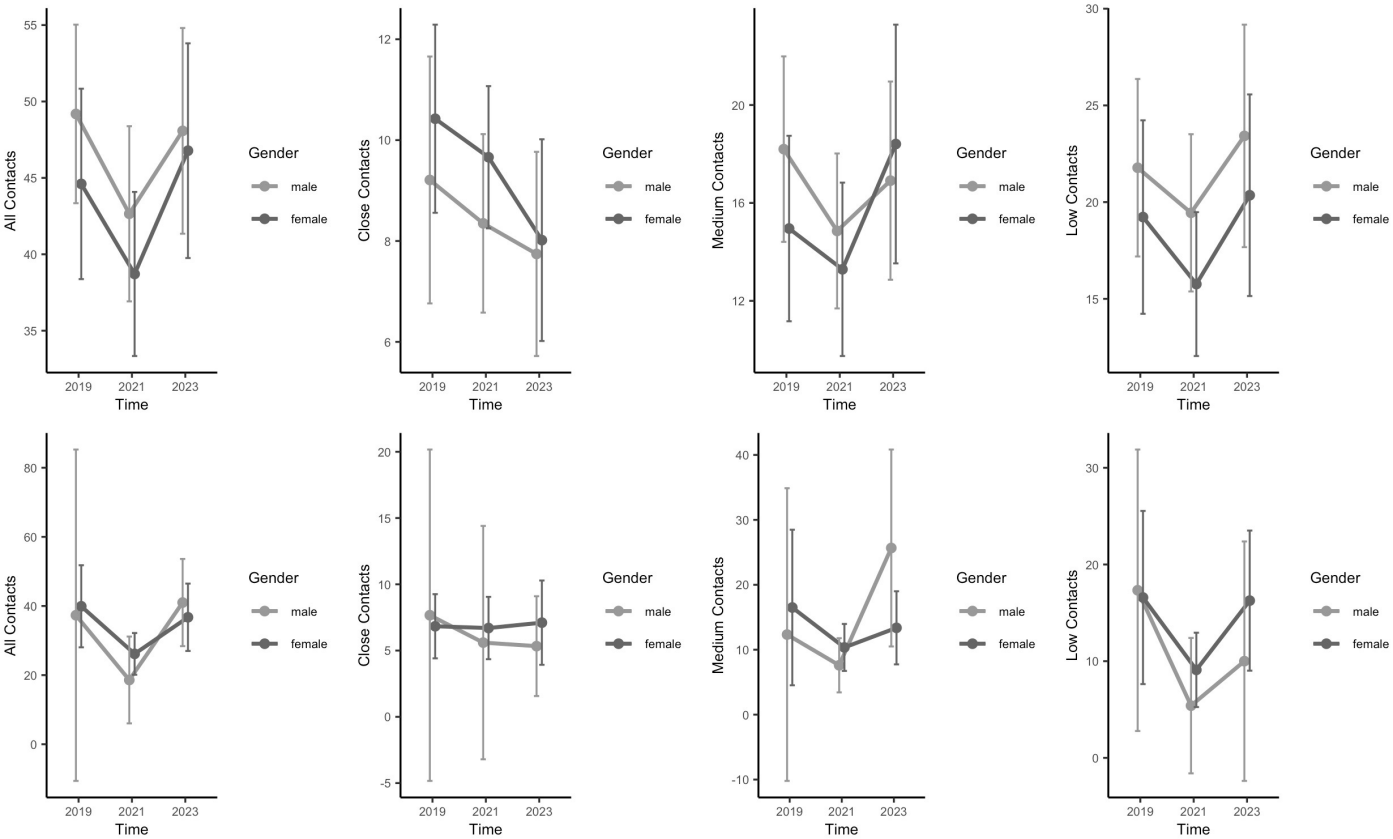
Overview over the number of social contacts for participants differing in their level of depression level (with and without) and gender. **(Top)** Participants without depression (*n*_*no*_*depression*_ = 158), **(Bottom)** participants with depression (*n*_*depression*_ = 37) over the three time points.

As described in our analytical approach, we used generalized linear mixed effects models with Poisson distribution to investigate how social contacts change over the COVID-19 pandemic. We included random intercepts for participants, and fixed effects for the time point of the pandemic, the subjective depression level (BDI-II, splitted in participants with vs. without depression), age and gender. For age, we used and median split (*Mdn* = 72 years) for categorization. For depression, we differentiated between depressive and non-depressive. For this, we used the cut-off criterion for mild depression (>14) and summarized all severity levels of depression under “depression” (in comparison to “no depression”). Contact_total_ was used as dependent variable. Table 3 shows estimated parameters for fixed and random effects of the model described above and their 95% confidence intervals for social contacts as dependent variable. Time was used as numerical variable. The results show significant effects of time point of the pandemic, depression level, as well as a three-way interaction between time point, depression level and gender. We also found significant two-way interactions (see Table 3). Figure 2 shows the plots for the three time points (before, during, after) the pandemic, depression level [no depression (upper row) vs. mild to severe depression (lower row)], gender, age (< 72 vs. ≥72).

**Table 3:** Estimated parameters for social contacts_total_.

**Fixed effects**	**Estimates**	**Std. error**	***z*-value**	**95% CI**
**Intercept**	**3.75**	**0.06**	**61.66**	**(3.64;3.88)**
Time	−0.02	0.01	−1.16	(−0.04;0.01)
**BDI-II**_**mild − to − severe**_	**−0.61**	**0.11**	**−5.56**	**(−0.83;** **−0.40)**
Gender_female_	−0.16	0.09	−1.89	(−0.34;0.01)
Age_≥72_	−0.03	0.03	−0.99	(−0.09;0.03)
**Time** **×BDI-II**_**mild − to − severe**_	**0.15**	**0.06**	**2.36**	**(0.03;0.27)**
**Time** **×Gender**_**female**_	**0.04**	**0.02**	**2.30**	**(0.01;0.08)**
**BDI-II**_**mild − to − severe**_ **×Gender**_**female**_	**0.57**	**0.13**	**4.43**	**(0.32;0.83)**
**Time** **×BDI-II**_**mild − to − severe**_ **×Gender**_**female**_	**−0.18**	**0.07**	**−2.50**	**(−0.33;−0.04)**
**Random effects**	**Variance**	**Std. dev**.		
Participant (Intercept)	0.30	0.54		

*N* = 169 participants. Time as continuous variable. BDI-II = Beck's depression inventory conducted the subjective depression level of a person; dichotomic split of persons without depression and with depression. For gender, we used male as reference category. For age, we used a median split (*Mdn* = 72 years). Significant parameter estimates are marked bold.

## 4 Discussion and implications

This longitudinal study addresses the change of social contacts in older adults because of the COVID-19 pandemic in south-west Germany. The results show a significant three-way interaction between time, sex, and level of depression [BDI-II, (28)]. First, with regard to the total number of contacts, there was a significant difference between participants with and without depression. As expected, depressed participants had significantly fewer social contacts that did not vary over time, regardless of the time point. In contrast, the Young-Old (< 72 years) who were not depressed showed a clear drop due to the pandemic, regardless of sex, while the opposite effect was seen for the Old-Old (≥72 years). For close social contacts in particular, there was a clear drop during the pandemic in all subjects who were not depressed, from which especially the Young-Old were unable to recover. In contrast, although the close social contacts among the depressed participants were significantly lower, they were also significantly more persistent and increased slightly over time. Our results do not take into account the duration of the illness (29, 30). As known from literature the Old-Old have fewer social contacts than the Young-Old. However, it appears that the Old-Old recover more quickly (30). In general, the present study makes important statements about how social contacts of older adults change over time. The increase in contacts among the Old-Old might be caused by family members and relatives taking care of this very vulnerable group and reactivating them. The Younger-Old (60–72 years) lost total and especially close contacts without reaching pre-pandemic levels. This might be caused by changes of contact behavior (less hand shaking, more physical distance) and/or increase of leisure activities with less social interactions (28). Further detailed analyses of the complex interplay of number of objective contacts and its type and loneliness will follow.

However, the present study also has some considerable limitations: As the TREND study did not include any surveys of the participants' social networks, participants' social networks in 2019 were surveyed retrospectively to have a baseline before COVID-19 pandemic. As the data analysis took longer than originally planned due to the restrictions during the pandemic, we had to change the reference month from May to November during data collection to prevent potential recall errors and gaps. In addition, we were no longer allowed to offer face-to-face appointments from December 2021 due to the increasing restrictions imposed by the Federal Ministry of Germany during the COVID-19 pandemic. For that reason, we had to switch the data collection format to a video condition. Considering the age of our participants, this worked surprisingly well. However, this change could result in a selection of participants who are familiar with computers and video conferencing. Another limitation of our study was that due to time constraints, we had to use the same reference month (November 2023) for the third time point (“after the pandemic”) for all subjects, including those who actually had May as their reference month. This could lead to the data not being comparable with the previous two time points, e.g., due to the different seasons (autumn vs. spring, which also entail different (leisure) activities). Our subjects were part of a cohort from an early detection study for neurodegeneration (TREND study), which could suggest that our sample had greater cognitive impairment than the general population. However, we also looked at data from neuropsychological tests [MMSE, (31)] collected at regular TREND study visits before the pandemic, during the pandemic and after the pandemic. These data show that the participants in our sample were in the normal range at all three time points (see Supplementary material) and showed no major cognitive impairment. This could be explained by the fact that participation in these additional surveys was voluntary and did not directly serve the TREND study objective, meaning that it was primarily highly motivated, above-average educated and cognitively fitter test subjects who took part. Furthermore, a recall bias would mean that periods further back in time are less well-remembered and, in the case of the networks, fewer contacts are reported for the period “before the pandemic”. However, we see in our social network data that the total number of social contacts decreases from “before the pandemic” to “during the pandemic” and then increases again at the time “after the pandemic”. With a recall bias, a continuous increase over the course of the study would have been more likely.

There are other studies that have investigated long term changes in the social networks of participants and recorded both the current state of the social ego-network and the state in the past on a single assessment visit (32–34). Some of the retrospective reference points for the social network in the past were even longer ago than in our study [up to 4 years, see (32)]. Like us in our study, the authors of these studies also see limitations due to the retrospective assessment. To our knowledge there are no systematic studies on the validity and reliability of self-reports in ego-networks in different age groups beside a small study with drug users (34). Forgetting seems to be decreased by behavioral specificity and salience (34). A recall bias cannot be in our study excluded, but in case of memory deficits for the real number of network partners during the reference period before COVID crisis this would even attenuate the pre-post difference and would not exaggerate it. Despite these limitations, this type of study provides a useful insight into the changes in social networks from a self-perspective: in our case, the changes in older people's ego-centered networks before, during and after the COVID-19 pandemic, which have not yet been investigated in other studies with this a large number of participants.

In summary, the present results provide new insights into the influence of social contacts on older adults during the COVID-19 pandemic. Overall, the pandemic and the level of personal depression seem to have a significant impact on the number of contacts people make. In particular, as many studies have already shown, the season within year and the medium of contact appear to have a decisive influence on this (35, 36). Nevertheless, the results show that, as expected, the number of contacts was increasing again after pandemic in the Old-Old but did not reach the pre-pandemic level in the Young-Old. The different trajectories between Young-Old and Old-Old could be due to the fact that relatives and friends are once again taking more care of this vulnerable very old group after the pandemic and are also actively approaching them, while the Young-Old (< 72 years) have to become active themselves in order to maintain their contacts or make new contacts and are less accustomed to this behavior since the pandemic (37–39). These trends suggest that it is not only the Old-Old who need special support, but the Young-Old. Close contacts stay on a lower level in all age groups after pandemic, even in the non-depressed group. Projects such as the loneliness barometer (10) therefore appear to be well-founded in order to prevent loneliness in old age.

## 5 Conclusions

In response to the study aims of examining the long-term effects of the COVID-19 pandemic on social contacts and wellbeing among older adults, this study reveals that pandemic-related social isolation had slightly differing impacts across age groups. While the Old-Old (≥72 years) regained some social interaction due to increased support from family and friends, all non-depressed subjects but especially Young-Old (< 72 years) struggled to recover pre-pandemic contact levels, loosing close relationships. Depression consistently correlated with reduced social engagement, highlighting the need for targeted support for older adults. The findings emphasize that younger seniors, particularly, could benefit from structured interventions to maintain social connections and prevent loneliness, validating the importance of ongoing initiatives like the loneliness barometer.

## Data Availability

The original contributions presented in the study are included in the article/Supplementary material, further inquiries can be directed to the corresponding author.
