# Supplementary material for: COVID-19 and social distancing: pandemic has altered social relationships and contacts in older adults over 4 years
Source: Front Public Health. 2024 Dec 16;12:1456829. doi: 10.3389/fpubh.2024.1456829 (PMC11683058; doi:10.3389/fpubh.2024.1456829)
Supplement: Supplementary file 1 [file Data_Sheet_1.zip › Supplementary Material /Supplement_CRF+Questionnaire.docx]

Social network analysis

The following questions refer to your social relationships and your social contacts. We use a special method of social research called “social network analysis”.

This method is based on the idea that people can have, establish, and lose connections with each other (such a connection is often described as an “invisible bond” that exists between people). There is a particularly strong connection to some people (friends, partners, colleagues, etc.), while there is only a weak connection to others (acquaintances, etc.). Every person therefore finds themselves in such a relationship structure of more or less important social contacts.

**Step-by-step guide to completing your social network circles**

1. List all the people and organizations (e.g. association/club or initiative) that play an important role in your social network on the “People in my network” sheet. This stays with you after your participation. Keep this carefully. You will need it again in future interviews.
2. You will now receive two social network circles.

The first network circle represents your current network at the time of the corona pandemic 2021. The second network circle represents your network before the outbreak of the coronavirus pandemic in 2019.

1.
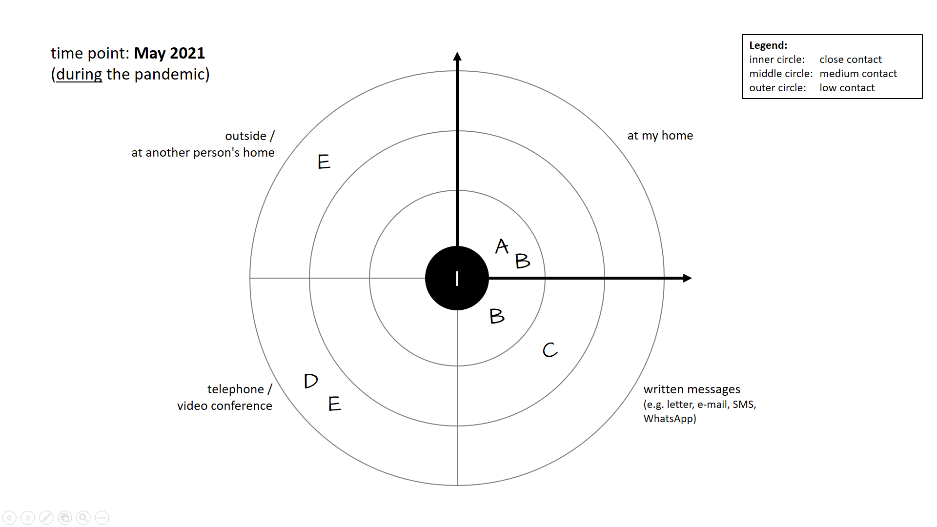
Take the network circle for 2021 and insert the people you have noted on the “People in my network” sheet into the circle scheme. Imagine that you form the center of the network (“I”). Draw close people closer to you and less close people further away.
2. When inserting people into the circle scheme, the type of contact should be considered: In which way or via which medium do you have contact with the respective person (in person at home, in person outside, by telephone/video telephony, virtually via messages such as SMS, WhatsApp, e-mail)?
3. Now reflect: Which social contacts did you have before the outbreak of the pandemic?

Take the network circle scheme for 2019 and insert the “people in my network” to the circle again. Think about how the relationship has changed over time.

“People in my network”

**List the people you consider important for your social network in the table below. For data protection reasons, this sheet of paper will remain with you after your study visit. Please keep this sheet in a safe place. You will need it again in future interviews.**

| person | name |
| --- | --- |
| **A** |  |
| **B** |  |
| **C** |  |
| **D** |  |
| **E** |  |
| **F** |  |
| **G** |  |
| **H** |  |
| **I** |  |
| **J** |  |
| **K** |  |
| **L** |  |
| **M** |  |
| **N** |  |
| **O** |  |
| **P** |  |
| **Q** |  |
| **R** |  |
| **S** |  |
| **T** |  |
| **U** |  |
| **V** |  |
| **W** |  |
| **X** |  |
| **Y** |  |
| **Z** |  |

**Would you like to add more people? Please use the back page.**

| person | name |
| --- | --- |
| **A1** |  |
| **B1** |  |
| **C1** |  |
| **D1** |  |
| **E1** |  |
| **F1** |  |
| **G1** |  |
| **H1** |  |
| **I1** |  |
| **J1** |  |
| **K1** |  |
| **L1** |  |
| **M1** |  |
| **N1** |  |
| **O1** |  |
| **P1** |  |
| **Q1** |  |
| **R1** |  |
| **S1** |  |
| **T1** |  |
| **U1** |  |
| **V1** |  |
| **W1** |  |
| **X1** |  |
| **Y1** |  |
| **Z1** |  |

**Have you listed all the people in your social network?**

**Please go back to the step-by-step guide to filling out your network circle.**


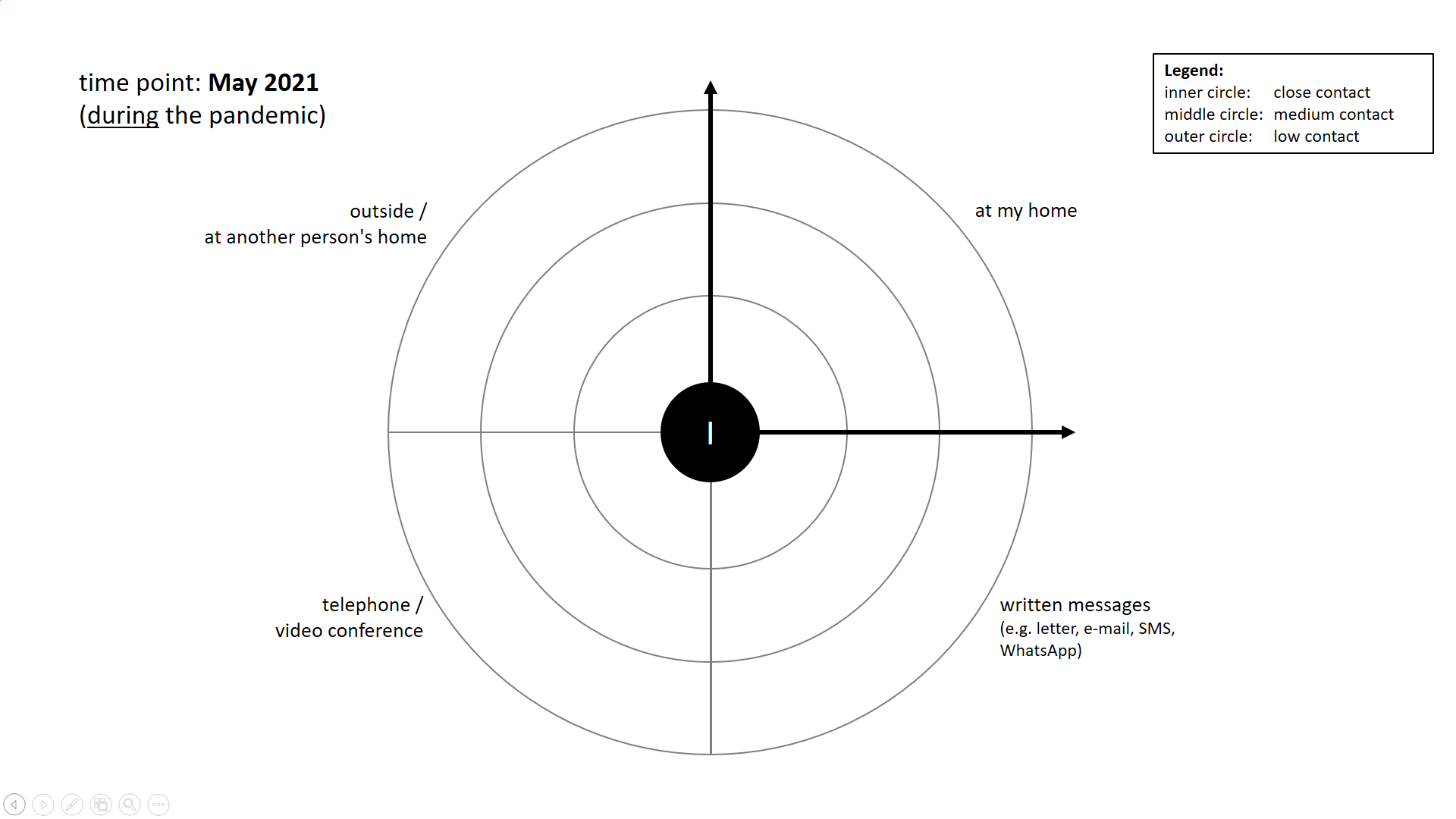


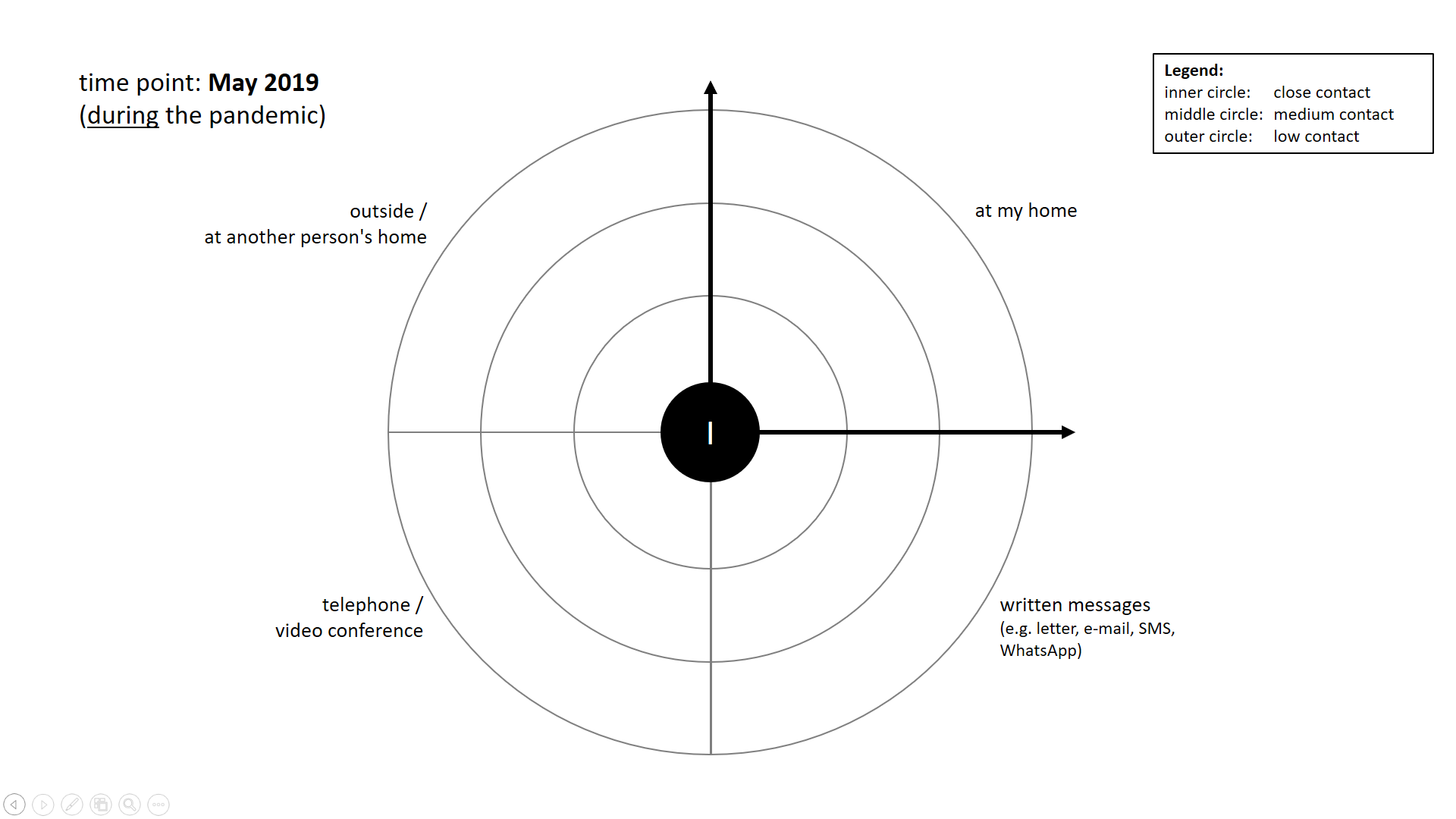


***This page and the following pages are filled in by the investigator during the interview.***

Some statistical information on the people in the participant’s network is recorded below (e.g. age, gender). To maintain anonymity, the persons are labeled “A”, “B” and “C”, etc.

| **Person** | **Age & Sex** | **How often did you have contact with the person in the reference month?** | **Type of relationship?**  *[Multiple answers allowed, underline the most applicable category]* | |
| --- | --- | --- | --- | --- |
| **A** | ____ [years]   - m - f | - daily - 4-6 x /week - 3-4 x/week - 1-2 x/week - 1-2 x/month - less often | - family & relatives - friends & acquaintances - neighbors - (former) colleagues | - professional helpers - club/association/initiative - leisure time - other |
|  | **Notes (e.g. type of family relationship):** | | | |
| **B** | ____ [years]   - m - f | - daily - 4-6 x /week - 3-4 x/week - 1-2 x/week - 1-2 x/month - less often | - family & relatives - friends & acquaintances - neighbors - (former) colleagues | - professional helpers - club/association/initiative - leisure time - other |
|  | **Notes (e.g. type of family relationship):** | | | |
| **C** | ____ [years]   - m - f | - daily - 4-6 x /week - 3-4 x/week - 1-2 x/week - 1-2 x/month - less often | - family & relatives - friends & acquaintances - neighbors - (former) colleagues | - professional helpers - club/association/initiative - leisure time - other |
|  | **Notes (e.g. type of family relationship):** | | | |
| **D** | ____ [years]   - m - f | - daily - 4-6 x /week - 3-4 x/week - 1-2 x/week - 1-2 x/month - less often | - family & relatives - friends & acquaintances - neighbors - (former) colleagues | - professional helpers - club/association/initiative - leisure time - other |
|  | **Notes (e.g. type of family relationship):** | | | |
| **E** | ____ [years]   - m - f | - daily - 4-6 x /week - 3-4 x/week - 1-2 x/week - 1-2 x/month - less often | - family & relatives - friends & acquaintances - neighbors - (former) colleagues | - professional helpers - club/association/initiative - leisure time - other |
|  | **Notes (e.g. type of family relationship):** | | | |
| **F** | ____ [years]   - m - f | - daily - 4-6 x /week - 3-4 x/week - 1-2 x/week - 1-2 x/month - less often | - family & relatives - friends & acquaintances - neighbors - (former) colleagues | - professional helpers - club/association/initiative - leisure time - other |
|  | **Notes (e.g. type of family relationship):** | | | |
| **G** | ____ [years]   - m - f | - daily - 4-6 x /week - 3-4 x/week - 1-2 x/week - 1-2 x/month - less often | - family & relatives - friends & acquaintances - neighbors - (former) colleagues | - professional helpers - club/association/initiative - leisure time - other |
|  | **Notes (e.g. type of family relationship):** | | | |
| **H** | ____ [years]   - m - f | - daily - 4-6 x /week - 3-4 x/week - 1-2 x/week - 1-2 x/month - less often | - family & relatives - friends & acquaintances - neighbors - (former) colleagues | - professional helpers - club/association/initiative - leisure time - other |
|  | **Notes (e.g. type of family relationship):** | | | |
| **I** | ____ [years]   - m - f | - daily - 4-6 x /week - 3-4 x/week - 1-2 x/week - 1-2 x/month - less often | - family & relatives - friends & acquaintances - neighbors - (former) colleagues | - professional helpers - club/association/initiative - leisure time - other |
|  | **Notes (e.g. type of family relationship):** | | | |
| **J** | ____ [years]   - m - f | - daily - 4-6 x /week - 3-4 x/week - 1-2 x/week - 1-2 x/month - less often | - family & relatives - friends & acquaintances - neighbors - (former) colleagues | - professional helpers - club/association/initiative - leisure time - other |
|  | **Notes (e.g. type of family relationship):** | | | |
| **K** | ____ [years]   - m - f | - daily - 4-6 x /week - 3-4 x/week - 1-2 x/week - 1-2 x/month - less often | - family & relatives - friends & acquaintances - neighbors - (former) colleagues | - professional helpers - club/association/initiative - leisure time - other |
|  | **Notes (e.g. type of family relationship):** | | | |
| **L** | ____ [years]   - m - f | - daily - 4-6 x /week - 3-4 x/week - 1-2 x/week - 1-2 x/month - less often | - family & relatives - friends & acquaintances - neighbors - (former) colleagues | - professional helpers - club/association/initiative - leisure time - other |
|  | **Notes (e.g. type of family relationship):** | | | |
| **M** | ____ [years]   - m - f | - daily - 4-6 x /week - 3-4 x/week - 1-2 x/week - 1-2 x/month - less often | ____ [years]   - m - f | - daily - 4-6 x /week - 3-4 x/week - 1-2 x/week - 1-2 x/month - less often |
|  | **Notes (e.g. type of family relationship):** | | | |
| **N** | ____ [years]   - m - f | - daily - 4-6 x /week - 3-4 x/week - 1-2 x/week - 1-2 x/month - less often | ____ [years]   - m - f | - daily - 4-6 x /week - 3-4 x/week - 1-2 x/week - 1-2 x/month - less often |
|  | **Notes (e.g. type of family relationship):** | | | |
| **O** | ____ [years]   - m - f | - daily - 4-6 x /week - 3-4 x/week - 1-2 x/week - 1-2 x/month - less often | ____ [years]   - m - f | - daily - 4-6 x /week - 3-4 x/week - 1-2 x/week - 1-2 x/month - less often |
|  | **Notes (e.g. type of family relationship):** | | | |
| **P** | ____ [years]   - m - f | - daily - 4-6 x /week - 3-4 x/week - 1-2 x/week - 1-2 x/month - less often | ____ [years]   - m - f | - daily - 4-6 x /week - 3-4 x/week - 1-2 x/week - 1-2 x/month - less often |
|  | **Notes (e.g. type of family relationship):** | | | |
| **Q** | ____ [years]   - m - f | - daily - 4-6 x /week - 3-4 x/week - 1-2 x/week - 1-2 x/month - less often | ____ [years]   - m - f | - daily - 4-6 x /week - 3-4 x/week - 1-2 x/week - 1-2 x/month - less often |
|  | **Notes (e.g. type of family relationship):** | | | |
| **R** | ____ [years]   - m - f | - daily - 4-6 x /week - 3-4 x/week - 1-2 x/week - 1-2 x/month - less often | ____ [years]   - m - f | - daily - 4-6 x /week - 3-4 x/week - 1-2 x/week - 1-2 x/month - less often |
|  | **Notes (e.g. type of family relationship):** | | | |
| **S** | ____ [years]   - m - f | - daily - 4-6 x /week - 3-4 x/week - 1-2 x/week - 1-2 x/month - less often | ____ [years]   - m - f | - daily - 4-6 x /week - 3-4 x/week - 1-2 x/week - 1-2 x/month - less often |
|  | **Notes (e.g. type of family relationship):** | | | |
| **T** | ____ [years]   - m - f | - daily - 4-6 x /week - 3-4 x/week - 1-2 x/week - 1-2 x/month - less often | ____ [years]   - m - f | - daily - 4-6 x /week - 3-4 x/week - 1-2 x/week - 1-2 x/month - less often |
|  | **Notes (e.g. type of family relationship):** | | | |
| **U** | ____ [years]   - m - f | - daily - 4-6 x /week - 3-4 x/week - 1-2 x/week - 1-2 x/month - less often | ____ [years]   - m - f | - daily - 4-6 x /week - 3-4 x/week - 1-2 x/week - 1-2 x/month - less often |
|  | **Notes (e.g. type of family relationship):** | | | |
| **V** | ____ [years]   - m - f | - daily - 4-6 x /week - 3-4 x/week - 1-2 x/week - 1-2 x/month - less often | ____ [years]   - m - f | - daily - 4-6 x /week - 3-4 x/week - 1-2 x/week - 1-2 x/month - less often |
|  | **Notes (e.g. type of family relationship):** | | | |
| **W** | ____ [years]   - m - f | - daily - 4-6 x /week - 3-4 x/week - 1-2 x/week - 1-2 x/month - less often | ____ [years]   - m - f | - daily - 4-6 x /week - 3-4 x/week - 1-2 x/week - 1-2 x/month - less often |
|  | **Notes (e.g. type of family relationship):** | | | |
| **X** | ____ [years]   - m - f | - daily - 4-6 x /week - 3-4 x/week - 1-2 x/week - 1-2 x/month - less often | ____ [years]   - m - f | - daily - 4-6 x /week - 3-4 x/week - 1-2 x/week - 1-2 x/month - less often |
|  | **Notes (e.g. type of family relationship):** | | | |
| **Y** | ____ [years]   - m - f | - daily - 4-6 x /week - 3-4 x/week - 1-2 x/week - 1-2 x/month - less often | ____ [years]   - m - f | - daily - 4-6 x /week - 3-4 x/week - 1-2 x/week - 1-2 x/month - less often |
|  | **Notes (e.g. type of family relationship):** | | | |
| **Z** | ____ [years]   - m - f | - daily - 4-6 x /week - 3-4 x/week - 1-2 x/week - 1-2 x/month - less often | ____ [years]   - m - f | - daily - 4-6 x /week - 3-4 x/week - 1-2 x/week - 1-2 x/month - less often |
|  | **Notes (e.g. type of family relationship):** | | | |

**What does your current social network look like?**

In the questionnaire on the following pages, we are now asking you again for certain information about the persons A to *[individualized information]* that you named to us in the survey on 04.02.2022. This concerns the **time period of November 2023.** If there are **other or new persons/groups in your social network in the meantime, you can also list these at the end of the questionnaire.**

In the areas highlighted in gray, you will find pre-printed information that we received from you in the last survey
(age, gender, type of relationship; if a group, how many group members).

**
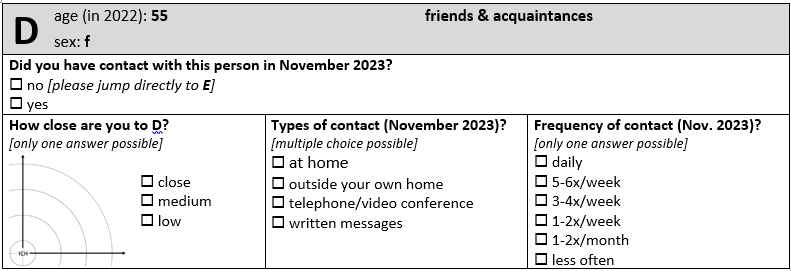
**

By **types of contact**, we are asking in which or **how many ways you had contact** with the person/group:

- At your home (receiving a visit, encounters in the hallway)?
- Did you meet "outside", i.e. outside your home (e.g. visits to other people's homes, outings, in a restaurant)?
- Have you spoken to each other on the phone or used video telephony (e.g. Zoom, Skype)?
- Have you sent each other written messages, e.g. via WhatsApp, text message, email, letter?

Here we ask about the **frequency of contact**:

🞎 daily

🞎 5-6x/week

🞎 3-4x/week

🞎 1-2x/week

🞎 1-2x/month

🞎 less often

This refers to **emotional closeness or how important this person is to you** or how connected you feel to a group, but not the frequency of contact.


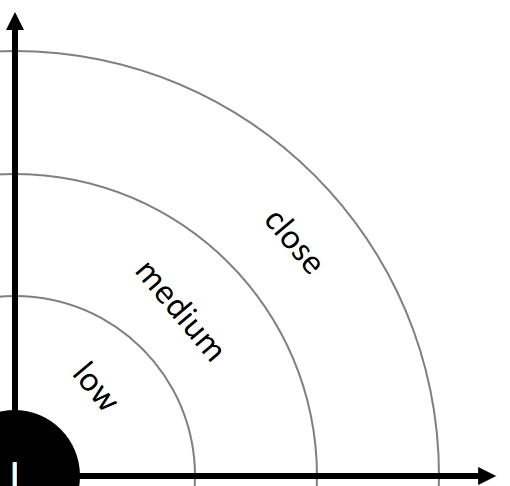


time period: **November 2023**

| **A** | age (in 2022): **47** | | | **friends & acquaintances** | |
| --- | --- | --- | --- | --- | --- |
|  | sex: **f** | | |  | |
| **Did you have contact with this person in November 2023?**  🞎 no *[please jump directly to* ***B****]*  🞎 yes | | | | | |
| **How close are you to A?** *[only one answer possible]* | | | **Types of contact (November 2023)?** *[multiple choice possible]* | | **Frequency of contact (Nov. 2023)?** *[only one answer possible]* |
| 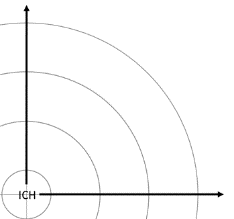 | | 🞎 close  🞎 medium  🞎 low | 🞎 at home  🞎 outside your own home  🞎 telephone/video conference  🞎 written messages | | 🞎 daily  🞎 5-6x/week  🞎 3-4x/week  🞎 1-2x/week  🞎 1-2x/month  🞎 less often |

| **B** | age (in 2022): **71** | | | **family** | |
| --- | --- | --- | --- | --- | --- |
|  | sex: **f** | | | *sister* | |
| **Did you have contact with this person in November 2023?**  🞎 no *[please jump directly to* ***C****]*  🞎 yes | | | | | |
| **How close are you to A?** *[only one answer possible]* | | | **Types of contact (November 2023)?** *[multiple choice possible]* | | **Frequency of contact (Nov. 2023)?** *[only one answer possible]* |
| 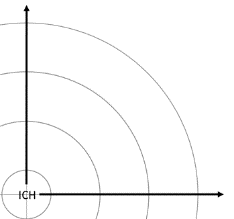 | | 🞎 close  🞎 medium  🞎 low | 🞎 at home  🞎 outside your own home  🞎 telephone/video conference  🞎 written messages | | 🞎 daily  🞎 5-6x/week  🞎 3-4x/week  🞎 1-2x/week  🞎 1-2x/month  🞎 less often |

| **C** | age (in 2022): **62** | | | **neighbors** | |
| --- | --- | --- | --- | --- | --- |
|  | sex: **f** | | |  | |
| **Did you have contact with this person in November 2023?**  🞎 no *[please jump directly to* ***D****]*  🞎 yes | | | | | |
| **How close are you to C?** *[only one answer possible]* | | | **Types of contact (November 2023)?** *[multiple choice possible]* | | **Frequency of contact (Nov. 2023)?** *[only one answer possible]* |
| 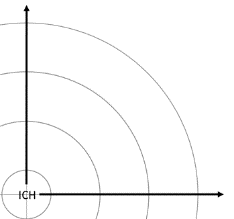 | | 🞎 close  🞎 medium  🞎 low | 🞎 at home  🞎 outside your own home  🞎 telephone/video conference  🞎 written messages | | 🞎 daily  🞎 5-6x/week  🞎 3-4x/week  🞎 1-2x/week  🞎 1-2x/month  🞎 less often |

| **D** | age (in 2022): **72** | | | **friends & acquaintances** | |
| --- | --- | --- | --- | --- | --- |
|  | sex: **m** | | |  | |
| **Did you have contact with this person in November 2023?**  🞎 no *[please jump directly to* ***E****]*  🞎 yes | | | | | |
| **How close are you to D?** *[only one answer possible]* | | | **Types of contact (November 2023)?** *[multiple choice possible]* | | **Frequency of contact (Nov. 2023)?** *[only one answer possible]* |
| 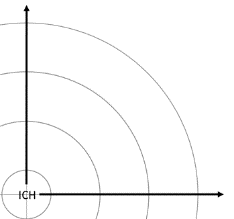 | | 🞎 close  🞎 medium  🞎 low | 🞎 at home  🞎 outside your own home  🞎 telephone/video conference  🞎 written messages | | 🞎 daily  🞎 5-6x/week  🞎 3-4x/week  🞎 1-2x/week  🞎 1-2x/month  🞎 less often |

*[analogous for all persons listed at the first two time points]*

**Were there other people/groups in your social network in November 2023 that were not previously mentioned in the questionnaire,** because they were not part of your social network in 2022?

If yes, **please provide information on these other persons in the following empty fields**. Only use as many fields as you need.

| **A1** | **age:** | | |  | |
| --- | --- | --- | --- | --- | --- |
|  | **sex:** | | |  |  |
| **Does A1 consist of several persons (e.g. married couple/group)?** 🞎 **no** 🞎 **yes**, A1 consists of approx. ____ persons | | | | | |
| **Where would you most likely classify this person/group?** *[only one answer possible]* | | **How close are you to A1?** *[only one answer possible]* | **Types of contact (November 2023)?** *[multiple choice possible]* | | **Frequency of contact (Nov. 2023)?** *[only one answer possible]* |
| - family & relatives - friends & acquaintances - neighbors - (former) colleagues - professional helpers - club/association/initiative - leisure time - other | | 🞎 close  🞎 medium  🞎 low | 🞎 at home  🞎 outside your own home  🞎 telephone/video conference  🞎 written messages | | 🞎 daily  🞎 5-6x/week  🞎 3-4x/week  🞎 1-2x/week  🞎 1-2x/month  🞎 less often |
| **Further information (e.g. type of relationship, what type of group, ...):** | | | | | |

| **B1** | **age:** | | |  | |
| --- | --- | --- | --- | --- | --- |
|  | **sex:** | | |  |  |
| **Does B1 consist of several persons (e.g. married couple/group)?** 🞎 **no** 🞎 **yes**, B1 consists of approx. ____ persons | | | | | |
| **Where would you most likely classify this person/group?** *[only one answer possible]* | | **How close are you to B1?** *[only one answer possible]* | **Types of contact (November 2023)?** *[multiple choice possible]* | | **Frequency of contact (Nov. 2023)?** *[only one answer possible]* |
| - family & relatives - friends & acquaintances - neighbors - (former) colleagues - professional helpers - club/association/initiative - leisure time - other | | 🞎 close  🞎 medium  🞎 low | 🞎 at home  🞎 outside your own home  🞎 telephone/video conference  🞎 written messages | | 🞎 daily  🞎 5-6x/week  🞎 3-4x/week  🞎 1-2x/week  🞎 1-2x/month  🞎 less often |
| **Further information (e.g. type of relationship, what type of group, ...):** | | | | | |

*[more empty fields for additional people in the social network]*
